# Supervised segmentation of phenotype descriptions for the human skeletal phenome using hybrid methods

**DOI:** 10.1186/1471-2105-13-265

**Published:** 2012-10-15

**Authors:** Tudor Groza, Jane Hunter, Andreas Zankl

**Affiliations:** 1 School of ITEE, The University of Queensland, Brisbane, Australia; 2 Bone Dysplasia Research Group, UQ Centre for Clinical Research (UQCCR), University of Queensland, Brisbane, Australia; 3 Genetic Health Queensland, Royal Brisbane and Women’s Hospital, Herston, Brisbane, Australia

## Abstract

**Background:**

Over the course of the last few years there has been a significant amount of research performed on ontology-based formalization of phenotype descriptions. In order to fully capture the intrinsic value and knowledge expressed within them, we need to take advantage of their inner structure, which implicitly combines qualities and anatomical entities. The first step in this process is the segmentation of the phenotype descriptions into their atomic elements.

**Results:**

We present a two-phase hybrid segmentation method that combines a series individual classifiers using different aggregation schemes (set operations and simple majority voting). The approach is tested on a corpus comprised of skeletal phenotype descriptions emerged from the Human Phenotype Ontology. Experimental results show that the best hybrid method achieves an F-Score of 97.05% in the first phase and F-Scores of 97.16% / 94.50% in the second phase.

**Conclusions:**

The performance of the initial segmentation of anatomical entities and qualities (phase I) is not affected by the presence / absence of external resources, such as domain dictionaries. From a generic perspective, hybrid methods may not always improve the segmentation accuracy as they are heavily dependent on the goal and data characteristics.

## Background

Phenotype descriptions are fundamentally important for our deeper understanding of genetics and evolutionary relationships. These facilitate the computation and analysis of evolutionary questions related to a varied range of issues, such as the genetic and developmental bases of correlated characters or the paleontological correlates of particular types of change in genes, gene networks and developmental pathways [1]. The literature contains a wealth of such phenotype descriptions, usually reported as free-text entries, similar to typical clinical summaries. A first and crucial step required to be able to take advantage of this knowledge is to model and capture them in a machine-processable format.

Over the course of the last few years there has been a significant amount of research performed on ontology-based formalization of phenotypes. The Mammalian Phenotype Ontology [2], the Human Phenotype Ontology (HPO) [3], the Elements of Morphology Project [4] – focusing on phenotypic variations of the head and face – or the Phenoscape Project [5] – aimed at representing and capturing phylogenetic studies on ostariophysan fishes – are some of the most representative projects in this area. Formalized phenotypic descriptions have then been successfully used for studying cross-species phenotype networks [6,7], linking human diseases to animal models [8] or predicting diagnoses using semantic similarity measures [9].

However, as noted also by Gkoutos et al. [10], in order to fully capture the intrinsic value and knowledge expressed by these descriptions, we require a more precise and fine-grained representation for them. Most phenotype terms implicitly combine anatomical entities with qualities. For example, HP:0010230 (*Cone-shaped epiphyses of the phalanges of the hand*) describes the anatomical entity *epiphyses of the phalanges of the hand* that bears the quality *cone-shaped*. Other terms represent atomic phenotypes that do not externalize directly this association, e.g., HP:0010884 (*Acromelia*), although their semantics can still be encoded using the same format, e.g., use the explicit meaning of *Acromelia* that denotes *shortness of the distal part of a limb*. This has led to the emergence of the Entity-Quality (EQ) formalism that enables the decomposition of phenotypic descriptions using ontologies, such as the Foundational Model of Anatomy (FMA) [11] – describing anatomical concepts and the Phenotype and Trait Ontology (PATO) [10] – comprising quality definitions. Subsequently, tools for manually creating such associations have been proposed, e.g., Phenoscape [5] and Phenex [12].

In this paper, we make the first step towards the automatic creation of decomposed phenotype descriptions by focusing on their initial segmentation, i.e., splitting a given phenotype term into chunks corresponding to anatomical entities and qualities. The second step required to complete this process would be the alignment of the resulting segments to ontological concepts.

The context of our research is provided by the SKELETOME project [13], which aims to create a community-driven knowledge curation platform for the skeletal dysplasia domain^a^. To date, we have developed an ontology, the Bone Dysplasia Ontology [14], capable of capturing associations between skeletal dysplasias, gene mutations and phenotypic descriptions, the latter grounded in HPO concepts. The decomposition of phenotype descriptions, in our case represented mostly by radiographic findings of the skeletal system, would enable a fine-grained exploration of the phenotype space, and hence the exploration of commonalities between disorders based on the anatomical localization of phenotypes and the development of anatomical localization - oriented decision support methods. Consequently, our work focuses on elements associated only with the human skeletal phenome.

The segmentation of skeletal phenotype descriptions raises a series of structural and semantic challenges. From a structural perspective, there are four classes of segments that need to be considered: qualities, qualifiers, anatomical coordinates and anatomical entities, the latter being decomposable into parts and sub-parts. Considering, for example, *irregular ossification of the proximal radial metaphysis*, *ossification* denotes the quality and has associated a qualifier (*irregular*), while *radial metaphysis* denotes the anatomical entity and has associated an anatomical coordinate (*proximal*). Secondly, due to the composite nature of the anatomical concepts, there is no uniform pattern that can be assumed for segmentation. For example, *epiphyseal widening of the hand phalanges* is the same as *broadening of the epiphyses of the phalanges of the hand*. From a semantic perspective, one challenge is provided by ambiguity, e.g., *irregular ossification of the proximal****radial ****metaphysis* vs. ***radial ****club hand*. Here, *radial* refers to the anatomical entity *radius* in the first case, and to an anatomical coordinate in the second case. Finally, the existing terminology contains metaphorical expressions that may pose issues for an accurate detection/classification, e.g., *bone-in-bone appearance* or *angel-shaped epiphyses*.

Machine learning methods have proved to be successful at dealing with the above mentioned challenges, although rule-based methods could also be employed with a high precision, but most likely at a trade-off of a lower recall. Conditional Random Fields (CRF) [15], in particular, have been reported to achieve good results both for segmentation tasks, as well as for classification tasks in the biomedical domain (see, for example, [16,17] or [18]). Recent works, however, has concentrated on using ensembles of classifiers (hybrid approaches) to overcome the issues associated with using single classifiers. As an example, the approaches described in [19] and [20] have used sets of classifiers (three by the former and six by the latter) aggregated via different voting schemes for gene/protein mention tagging.

**Figure 1:**
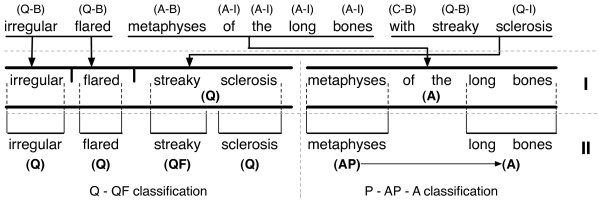
**Example of segmentation of a phenotype description according to the two phases of our approach.** In phase I, tokens are labeled using BIO labeling corresponding to each class: Q (quality), A (anatomy), C (connective). Label O (not present in the figure) is used to denote tokens outside the target classes. Similarly, in phase II, specific classifiers are used to segment quality-qualifier pairs (Q-QF) and anatomical parts (A), sub-parts (AP) and coordinates (P).

Our solution also relies on training divergent models via an ensemble of classifiers and aggregating the results via set operations or simple majority voting. More concretely, we propose a two-phase process, as exemplified in Figure 1, that first segments the input into coarse qualities and anatomical entities, then re-orders them according to their class and finally splits each resulting segment into its atomic parts. These atomic elements correspond to quality-qualifier pairs (e.g., *streaky* - *sclerosis* in our example), anatomical coordinate - anatomical concept associations and part-sub part relationships between anatomical concepts (e.g., *metaphyses* - of - *long bones* in Figure 1). Our ensemble of classifiers comprises two CRFs and two Support Vector Machines (SVM)-based chunkers. We perform an extensive series of experiments to find the optimal aggregation strategy for the ensemble, i.e., the strategy the maximises the segmentation performance.

## Methods

As mentioned previously, our solution proposes a two-phase process. Within each phase we train the ensemble of classifiers to achieve the corresponding segmentation goals. In this section, we discuss the corpus used for training and testing, the complete set of features used for segmentation, as well as the different aggregation strategies. The following section presents the segmentation efficiency achieved by each classifier, via a ten-fold cross-validation with stratification.

### Phenotype description corpora

As a first step, we have created a corpus comprising a total of 3,742 phenotype descriptions by listing all children under HP:0000924 (*Abnormality of the skeletal system*). We could have also collected such descriptions from the literature, however working directly with terms that denote concepts in one of the most widely used phenotype ontologies (like HPO) will bring us closer to achieving the overall goal of proving an automatic mechanism for the conceptual decomposition of phenotype ontologies. At the same time, given the context provided by the SKELETOME project, we are particularly interested in segmenting HPO concepts, since they will represent our input data when building anatomical localization-oriented decision support methods.

This corpus has been manually annotated using the BIO labelling scheme, where ’B’ stands for the beginning of a concept, ’I’ for inside a concept and ’O’ for outside any concept. In practice, the scheme has been used in association with the target classes for phase I, i.e., anatomy and quality. More concretely, we have used the following labels (some exemplified in Figure 1): (i) A-B/A-I, beginning of and inside an anatomical concept (e.g., *metaphyses of the long bones* in Figure 1); (ii) Q-B/Q-I, beginning of and inside a quality concept (e.g., *irregular*, *flared* and *streaky sclerosis* in Figure 1); (iii) C-B/C-I, beginning of and inside a connective element (i.e., conjunction – *with* in Figure 1); (iv) O, outside all classes of interest. Table 1 provides an overview of the distribution of types of concepts (and associated labels) in the corpus. The corpus is available at: http://purl.org/skeletome/corpora/HPTagged.

**Table 1:** Phenotype description corpus statistics

**Label category**	**Count**	**Percentage**
Anatomy (A-B/A-I)	13,003	70.08%
Quality (Q-B/Q-I)	5,465	29.45%
Connectives (C-B/C-I)	43	0.23%
Outside (O)	45	0.24%
TOTAL	18,556	

The above listed target classes (or labels) have emerged directly from the EQ syntax, and as such, provide a 1-to-1 mapping between the output of phase I classifier and possible EQ statements. As described in [12], EQ associates a concept from an organism-specific ontology (e.g., FMA:46565 – *Skull*) with a quality term from PATO. The latter describes the quality or value of some attribute of the entity, e.g., shape (*rectangular*), size (*large*), etc. As it can be observed our labels A and Q represent the Entity and Quality counterparts in the EQ syntax.

On the other hand, as mentioned in the Introduction, not all phenotype descriptions externalise in a direct manner the Entity-Quality relationship, for example, HP:0010884 (*Acromelia*) or HP:0000943 (*Dysostosis multiplex*). We call such phenotype descriptions *atomic phenotypes* and they represent, in principle, terms that denote distinctive qualities associated to vaguely specified anatomical entities (e.g., *distal part of a limb* in the case of *Acromelia*) or more complex concepts, such as processes or qualities of qualities. These terms require manual decomposition (since their meaning is implicit not explicit), and thus cannot be directly segmented into their elementary anatomic and quality concepts. Within our annotated corpus, atomic phenotypes have been labeled as qualities (i.e., Q-B/Q-I), because from our perspective they bear a qualitative purpose. This no longer conforms to the EQ syntax, since atomic phenotypes are not qualities per se and hence they do not exist in PATO. However, our aim is to segment phenotype descriptions into a format that can enable further processing (e.g., for ontology population) and not to achieve 100% conformance to EQ. Furthermore, this labelling enables us to continue the decomposition process in phase II with finding possible qualifier – quality associations, e.g., *severe* – *coxa vara*.

In the second step, we have processed the resulting annotated corpus to create two corpora relevant for phase II, i.e., a quality corpus and an anatomy corpus. Each of these corpora contains unique entries, hence eliminating the duplicates that may be present in the original phenotype descriptions. More concretely, terms that appear several times as a result of the segmentation in phase I (e.g., *phalanx of finger* or *short*) are retained only once. The set of labels used within the two corpora are described below.

The goal of the anatomy segmentation in the second phase is to identify part – subpart relationships between anatomical concepts resulted from the initial segmentation. In the example listed in Figure 1, the phase I segmentation will identify the anatomical entity *metaphyses of the long bones*. Phase II anatomical segmentation processes further this entity and aims to identify the main anatomical concept, i.e., *long bones* and its subparts – in this case *metaphyses*. In addition to these, we also aim to find anatomical coordinates, such as *proximal*, *lateral*, etc. (e.g., *proximal* metaphyses of the long bones). The Anatomy corpus uses the following labels: (i) A-B/A-I, beginning of and inside a main anatomical concept (e.g., *long bones* in Figure 1); (ii) AP-B/AP-I, beginning of and inside an anatomical part concept (e.g., *metaphyses* in Figure 1); (iii) PB, an anatomical coordinate (e.g., *proximal*) - as a remark anatomical coordinates are usually identified by single tokens and consequently we have not used additional labels; if multiple anatomical coordinates are present, they are identified individually and connected via conjunctive tokens; (iv) C-B/C-I, beginning of and inside a connective token (e.g., *of the* in Figure 1); (v) O, outside all classes of interest.

Similarly, the goal of the quality segmentation in this second phase is to identify qualifier – quality associations, e.g., *streaky* – *sclerosis* in Figure 1. The quality corpus hence uses the following labels: (i) Q-B/Q-I, beginning of and inside a quality concept (e.g., *irregular*, *flared*, *sclerosis* in Figure 1); (ii) QF-B/QF-I, beginning of and inside a qualifier concept (e.g., *streaky* in Figure 1); (iii) C-B/C-I, beginning of and inside a connective token; (iv) O, outside all classes of interest.

The above described corpora are available at http://purl.org/skeletome/corpora/HPAnatomy (the anatomy corpus) and http://purl.org/skeletome/corpora/HPQualities (the qualities corpus). Finally, Tables 2 and 3 present the distribution of the types of entities (and associated labels) present in the Anatomy and Quality corpora.

**Table 2:** Statistics for the Anatomy corpora used in phase II

**Label category**	**Count**	**Percentage**
Main anatomy (A-B/A-I)	2,984	41.01%
Anatomy part (AP-B/AP-I)	1,209	16.61%
Anatomy coordinate (PB)	698	9.60%
Connectives (C-B/C-I)	2,354	32.35%
Outside (O)	31	0.43%
TOTAL	7,276	

**Table 3:** Statistics for the Quality corpora used in phase II

**Label category**	**Count**	**Percentage**
Quality (Q-B/Q-I)	2,141	76.31%
Qualifier (QF-B/QF-I)	590	21.03%
Connectives (C-B/C-I)	52	1.84%
Outside (O)	23	0.82
TOTAL	2,806	

### Classifier features

The sets of features used by the classifiers, individually and within each phase, are in principle almost the same with a few exceptions: 

• Within each phase, in order to produce divergent classifiers, we use different window and n-gram sizes (for prefix and suffix) and include or omit the domain dictionaries (i.e., Anatomy and Quality), and

• Within the second phase, we omit completely the domain dictionaries.

In addition to local token-based features, we use three external resources: (i) the Foundational Model of Anatomy Ontology, (ii) the Phenotype and Trait Ontology, and (iii) the SPECIALIST (LexAccess) lexicon [21], which is a syntactic lexicon of biomedical and general English that records for each lexical item (word or term) the syntactic, morphological, and orthographic information.

The local features used by the classifiers are described below. All examples associated with the definitions use the phenotype description from Figure 1 and consider *metaphyses* to be the token in focus. Also, for clarification purposes, the symbol @ represents the absence of a token at the corresponding position. 

•**Token** – The token currently in focus. Example: *metaphyses*

•**Token lemma (linguistic)** – The linguistic stem of the token. Example: *metaphyse*

•**Token base (LexAccess)** – The base of the token as defined in LexAccess. Example: *metaphysis*

•**Token POS tag (linguistic)** – The linguistic part of speech tag. Example: *NNP*

•**Token category (LexAccess)** – The token category as defined in LexAccess. Example: *noun*

•**Token shape** – All capitalized letters are replaced by ’A’, all non-capitalized letters are replaced by ’a’, all digits are replaced by ’0’, the rest of the types of characters remain unaltered. Example: *aaaaaaaaaa*

•**Token brief shape** – The compressed version of the token shape created by suppressing all consecutive equal symbols. Example: *a*

•**Unigram context** (variable window size) – Unigram of the token and surrounding context. Example for window size 3: *@ irregular flared metaphyses of the long*

•**Bigram context** (variable window size) – Bigrams formed by the token and the surrounding context. Example for window size 3: *@-irregular irregular-flared flared-metaphyses metaphyses-with with-streaky streaky-sclerosis*

•**Trigram context** (variable window size) – Trigrams formed by the token and the surrounding context. Example for window size 3: *@-irregular-flared irregular-flared-metaphyses flared-metaphyses-with metaphyses-with-streaky with-streaky-sclerosis*

•**Morphological feature: punctuation** – Values: punct / no depending on whether the token ends with a punctuation sign. Example: *no*

•**Morphological feature: digits** – All digits in the token are replaced by ’*’. Example: no*

•**Morphological feature: vowels** – All characters of the token except vowels are replaced by ’-’. Example: *-e-a—-e-*

•**Token prefix** (variable size) – Token prefixes of variable length. Example for size 5: *m me met meta metap*

•**Token suffix** (variable size) – Token suffixes of variable length. Example for size 5: *s es ses yses hyses*

The next set of features are dictionary-based. Their value signals the presence of the given input in the corresponding dictionary. Among the six dictionaries we use, two are domain dictionaries and have been built from FMA and PATO, respectively. 

•** Conjunctions** – Tokens denoting conjunctions. Example: *and, or*

•** Connections** – Tokens denoting connecting cue-phrases. Example: *in, at, of*

•** Coordinates** – Tokens denoting anatomical coordinates. Example: *central, left*

•** Ordinals** – Gazetteer consisting of ordinals. Example: *1st, 2nd*

•** Anatomy** – Gazetteer comprising FMA anatomical concepts. Example: *Epiphysis, Limb*

•** Qualities** – Gazetteer comprising PATO qualities. Example: *short, long*

Finally, the last set of features represent combinations of basic and dictionary-based features. Again, similar to the listing above, all examples associated with the definitions are from Figure 1 and consider *metaphyses* to be the token in focus. 

•** Token base (LexAccess) + Anatomy dictionary** – Signals the presence of the base of the token in the anatomy dictionary. Example: *anat*

•** Token base (LexAccess) + Quality dictionary** – Signals the presence of the base of the token in the quality dictionary. Example: *no*

•** Unigram context (variable window size) + Anatomy dictionary** – Unigram-based context signalling the presence of the tokens in the anatomy dictionary. Example for window size 3: *no no no no no no no*

•** Unigram context (variable window size) + Quality dictionary** – Unigram-based context signalling the presence of the tokens in the quality dictionary. Example for window size 3: *no no qual no no no qual*

•** Bigram context (variable window size) + Anatomy dictionary** – Bigram-based context signalling the presence of the tokens in the anatomy dictionary. Example for window size 3: *no-no no-no no-no no-no no-no no-no*

•** Bigram context (variable window size) + Quality dictionary** – Bigram-based context signalling the presence of the tokens in the quality dictionary. Example for window size 3: *no-no no-qual qual-no no-no no-no no-qual*

•** Unigram context (variable window size) + Token base (LexAccess)** – Unigram-based context formed by token bases from LexAccess. Example for window size 3: *@ irregular flare metaphysis of the long*

•** Unigram context (variable window size) + Token base (LexAccess) + Anatomy dictionary** – Unigram-based context signalling the presence of the tokens bases from LexAccess in the anatomy dictionary. Example for window size 3: *no anat no anat no no no*

•** Unigram context (variable window size) + Token base (LexAccess) + Quality dictionary** – Unigram-based context signalling the presence of the tokens bases from LexAccess in the quality dictionary. Example for window size 3: *no qual no no no no qual*

As opposed to most segmentation / named entity extraction approaches, we do not perform any post-processing. The only intermediary processing element we include is in between phases when we re-order and join (if necessary) the segments according to their class. As presented in Figure 1, at the end of phase I, individual tokens are labelled with their corresponding class, e.g., Q - quality and A - anatomy. Hence, before running the specific segmentation in phase II, we join the consecutive tokens that belong to the same entity. For example, the tokens *streaky* (Q-B), *sclerosis* (Q-I), are joined into a corresponding monolithic quality entity, which is then segmented in phase II.

### Divergent classifiers

For the experiments discussed in the next section, we have used three toolkits and adopted different training algorithms to produce four classifiers. These toolkits are: 

• CRF++ ( http://crfpp.googlecode.com/): a freely available CRF package. We have used it to train a forward parsing model;

• MALLET [22]: another freely available CRF package. We have used it to train both a forward, as well as a backward parsing model, however we then used only the forward parsing model because of its increased accuracy;

• YamCha [23]: a chunking package that uses SVM classification. We have trained two models that differ in the method used for the multi-class classification, i.e., one vs. one or one vs. all.

### Algorithm 1 Simple majority voting scheme

1: **for***T*_*i *_∈* input ***all**

2: *V*_*i *_(*X*) =* vote*_*X *_(*T*_*i*_), where *X *∈ {CRF++,

MALLET, YAMCHA1VS1,

YAMCHA1VSALL }

3: 

4:**if ***V*_*i*_(*X*) is the same for any 3 methods *X ***then**

5: return *V*_*i*_(*X*)

6: **end if**

7: 

8: **if ***V*_*i*_(*X*) is the same for any 2 methods *X*

AND the other 2 methods are in

contradiction **then**

9: return *V*_*i*_(*X*)

10: **end if**

11: 

12: **if ***V*_*i*_(*X*) is the same for any 2 methods *X*

AND the other 2 methods agree **then**

13: return VETO

14: **end if**

15: 

16: **if ***V*_*i*_(*X*) is different for any method *X ***then**

17: return VETO

18: **end if**

19: **end for**

### Aggregation strategies

In addition to individual classifier-based experiments, we have also treated them as an ensemble and used two types of strategies for aggregating the classification / segmentation results: (i) set operations, and (ii) simple majority voting.

The set operations used within our experiments have been union and intersection. Union assumes the results of both inputs to be correct. As opposed to a single classifier, union will have the tendency to increase the recall at the expense of the precision. On the other hand, intersection will consider only the common results to be correct, which will, obviously, increase precision and lower the recall. As detailed in the next section, we have used different strategies for combining these set operations.

In the case of simple majority voting, the intuition is that the results commonly produced by most classifiers should be the correct one. Alg. 1 lists the pseudo-code of the voting mechanism we have used. In principle, if two or more classifiers agree on a label then consensus is being reached. However, because we have a even number of classifiers, we have introduced also a veto vote for those cases in which no consensus is being reached by the classifiers. Experiments have been performed with the veto vote assigned to different classifiers.

## Results

All the experiments detailed below have been carried out on the corpora introduced in the previous section. We performed a ten-fold cross validation with stratification and averaged the results. The evaluation results of each phase are listed in individual tables in the Additional file 1.

### Phase I

The classifiers trained in phase I have used all features previously described with diverse window (1 to 3) and n-gram (3 to 5) sizes. In order to track the influence of the domain dictionaries on the segmentation results, we ran two sets of experiments: one with and one without the support of these dictionaries (and thus, of the other features that depend on them).

#### Individual classifier results

Results of the individual classifiers are presented in Tables S1 and S2 in the Additional file 1. We can observe that YamCha, in the 1 vs. All setting, constantly outperforms all the classifiers in both categories, although the difference is minimal. Also, it is interesting to observe that the segmentation results are hardly affected by the absence of the domain dictionaries, since there is a constant gap in performance of only 0.18%.

#### Set operations-based results

Tables S3 and S4 in the Additional file 1 present the top results when aggregating the individual segmentation via set operations. Here, we have used the two basic types of set operations (i.e., union and intersection) in two different settings: (i) paired aggregation, testing the joint performance of pairs of classifiers, and (ii) combined aggregation, testing the performance of combined paired aggregations.

In the first setting (paired aggregation), as expected, the top performances have been achieved by pairs that contained the YamCha classifier. The aggregation of the top two individual classifiers led to the best performance in this group, i.e., 96.85% (with domain dictionaries) and 96.70% (without) for YamCha1vs1 ∪ YamCha1vAll . The effect of the union operation can be clearly seen in the results, since the combined precision is lower than the individual precision of the two classifiers, while the recall has improved. This was particularly the case for the MALLET ∪ YamCha1vsAll pair when using domain dictionaries and the CRF++ ∪ YamCha1vsAll when omitting domain dictionaries.

The second setting shows once more the superiority of the YamCha classifiers. While in the previous setting the paired aggregation of each YamCha classifier with a different one did not lead to the top performance, here this pairing has a positive effect. In practice, the union in each bracket gathers the best results of the two classifiers (YamCha complementing in principle the gaps left by its pair), with the final intersection pruning some of the false positives. The top result (96.91%) is almost identical to the top performance achieved by an individual classifier, the main difference being in the increased recall and lowered precision (as explained, due to the union operation).

#### Voting-based results

The final experiment in this phase used the voting mechanism to aggregate the results. As presented in the previous section, we opted for a simple majority with veto, i.e., the final result is the one voted by at least to classifiers with the remark that the other two need to disagree. The veto option is used in two cases: (i) when there is absolutely no consensus among classifiers, i.e., each classifier votes for a different label, and (ii) in the case of a tie between pairs of classifiers.

The results of the voting mechanism are presented in Tables S5 and S6 in the Additional file 1. The first remark that needs to be made is that, in both categories – with and without domain dictionaries – this mechanism constantly outperforms all the other strategies. Voting with YamCha1vsAll as veto owner in the dictionary-based category and YamCha1vs1 or YamCha1vsAll in the other category obtain the best performances, i.e., 97.05% and 96.90%, respectively. This result is, to some extent, expected since YamCha1vsAll was the best individual performer.

### Phase II

The specific classifiers trained for phase II did not use the domain dictionaries (i.e., Anatomy and Quality) nor the combined features that required them. In addition, for the quality classifiers we have also left out the Coordinates and Ordinals dictionaries. The experiments have followed the same structure used within phase I, i.e., we’ve tested the individual performance for each specific class, then the set operations and finally the voting mechanism.

Tables S7 and S8 in the Additional file 1 list the performances achieved by the individual classifiers. In the Anatomy category, CRF++ has outperformed the other classifiers with 97.11% F-score, followed by YamCha1vsAll (96.94%), which on the other hand, has achieved the best score in the Quality category (i.e., 94.50%). While here we can see a series of differences when compared to phase I (e.g., CRF++ achieves the best score, or the gap in performance in the Quality category is bigger than in any other case), the results of the set operations (see Tables S9 and S10 in the Additional file 1) are fairly close to the ones in phase I. It can be observed that all aggregations involving the YamCha classifier achieve the best results in both categories, the slight distinction being the best performer in the Anatomy category, i.e., CRF++ ∪ YamCha1vs1 – this is explained by the high individual results obtained by CRF++. A similar tendency can also be observed in the voting mechanism (Tables S11 and S12 in the Additional file 1), which again is the best of the three strategies and where the best results (97.16% and 94.50%, respectively) are achieved with CFR++ and MALLET owning the veto vote in the Anatomy category and YamCha1vsAll in the Quality category, respectively.

## Discussion

The experiments described in the previous section lead to four major conclusions. Firstly, as expected, hybrid methods are influenced by the individual performance of the classifiers chosen for aggregation. Secondly, on the positive side of things, such methods can, in practice, exploit the diversity and consistency among different classifiers to make final decision as opposed to single classifiers. In our case, for all types of segmentation in phase I and II (see Tables 4, 5, 6 and 7), the voting method has worked best due to its simple counting approach, while the set operations were penalised because of the ordering of the classifiers and of the operations used. However, this is not always the case. For example, in the gene mention tagging approach described in [24], all hybrid methods have outperformed the single classifiers. Hence, the efficiency and applicability of such hybrid approaches needs to be considered on a per use-case basis.

**Table 4:** Comparative segmentation results for phase I, with domain dictionaries

**Method**	**P (%)**	**R (%)**	**F-1 (%)**
Individual (YamCha1vsAll)	96.98	96.98	96.98
Set operations	96.64	97.17	96.91
Voting (veto: YamCha1vsAll)	97.05	97.05	**97.05**

**Table 5:** Comparative segmentation results for phase I, without domain dictionaries

**Method**	**P (%)**	**R (%)**	**F-1 (%)**
Individual (YamCha1vsAll)	96.80	96.80	96.80
Set operations	96.31	97.11	96.70
Voting (veto: YamCha1vs1)	96.90	96.90	**96.90**

**Table 6:** Comparative segmentation results for phase II on the Anatomy category

**Method**	**P (%)**	**R (%)**	**F-1 (%)**
Individual (CRF++)	97.11	97.11	97.11
Set operations	96.71	97.38	97.04
Voting (veto: CRF++ / MALLET)	97.16	97.16	**97.16**

**Table 7:** Comparative segmentation results for phase II on the Quality category

**Method**	**P (%)**	**R (%)**	**F-1 (%)**
Individual (YamCha1vsAll)	94.50	94.50	**94.50**
Set operations	93.84	94.64	94.24
Voting (YamCha1vsAll)	94.50	94.50	**94.50**

Thirdly, while the results presented throughout the entire set of experiments are not directly comparable, due to the difference in task and data characteristics, they do reveal that within the context provided by our segmentation goals YamCha is the most consistent classifier, and it should be always considered as foundation for any ensemble. Fourthly, for the initial segmentation of anatomical entities and qualities, domain dictionaries, and hence external resources, are not required. As shown in the experimental results, the difference in performance between the best approaches with and without domain dictionaries is minimal (around 0.15%), while from a positive perspective their absence reduces by half the feature set.

In order to get a deeper insight into the behaviour of the classifiers we have looked at their individual errors and at the label-based segmentation efficiency. When analysing the individual errors made by the classifiers, it was interesting to observe that each package had the tendency to make a certain type of error, usually different than that of the others. MALLET, for example, in phase I had a higher rate of error (76%) in choosing the generic class (i.e., Q was labeled as A and vice-versa), while CRF++ had issues mostly within the context of a generic class, in distinguishing the BIO labels, i.e., Q-I was labeled as Q-B, or A-B was labeled as A-I (in 81% of the cases). Finally, YamCha’s errors seemed to be uniformly distributed between the two previously mentioned types.

Tables 8, 9 and 10 list the label-based segmentation results and coverage for both phases. Overall, it can be observed that the classification results are not homogeneous, yet the F-1 scores meet our expectations. In the phase I corpus, in addition to having fairly distinct characteristics, the coverage of Q-B, A-B and A-I sums up to more than 90% of the labels, which leads to very good segmentation results. Subsequently, it is not surprising to observe that labels with a coverage of less than 1% of the annotated corpus have very low F-1 scores. The tokens labeled with O may also induce ambiguity in respect to both A-B/A-I, as well as C-B/C-I. They usually represent additional (temporal) information about the on-set of the phenotype (e.g., *in first year*) and contain elements ambiguous elements, such as, *in* – which is usually a connective token or *first* – which is usually found in anatomical concepts (e.g., *first metatarsal*). Similar results on this label can also be observed in the Anatomy and Quality corpora in phase II (Tables 9 and 10), achieving a lowest F-1 score of 0% (i.e., 0% precision, 0% recall) in the Anatomy corpus – here this label denotes extremely rarely found descriptive tokens, such as *esp.* or *region*. On the positive side, we can observe a consistent behaviour of the rest of the labels in the Anatomy corpus. This is because skeletal anatomical concepts have a fairly well-established part – subpart intrinsic structure that can only be exploited once they are delimited from the quality aspects of the phenotype descriptions. Finally, there are two remarks that are worth noting w.r.t. the Quality corpus: (i) the QF-I label has achieved a good score considering its coverage; the main issue here is ambiguity, as QF-I tokens are usually part of an enumeration of qualifiers, which can also act as QF-B tokens in most of the other cases; (ii) the Q-I label has also achieved a very good score considering its coverage, however, in this case, the score may be heavily supported by atomic phenotypes; in the vast majority of cases qualities are denoted by single tokens, and the presence of multiple quality tokens is usually associated with an atomic phenotype.

**Table 8:** Label-based segmentation results for phase I, including the coverage of the label

**Label**	**Coverage (%)**	**Average F-1 (%)**
Q-B	20.83	96.40
Q-I	8.62	88.93
A-B	15.17	94.91
A-I	54.91	98.79
C-B	0.22	47.73
C-I	0.01	10.00
O	0.24	12.50

**Table 9:** Label-based segmentation results for phase II, the Anatomy category, including the coverage of the label

**Label**	**Coverage (%)**	**Average F-1 (%)**
A-B	24.90	96.17
A-I	16.11	95.19
AP-B	15.67	96.10
AP-I	0.94	84.36
P-B	9.60	97.06
C-B	18.04	98.48
C-I	14.31	100.00
O	0.43	0.00

**Table 10:** Label-based segmentation results for phase II, the Quality category, including the coverage of the label

**Label**	**Coverage (%)**	**Average F-1 (%)**
Q-B	68.48	96.51
Q-I	7.83	79.06
QF-B	20.31	91.36
QF-I	0.72	55.62
C-B	1.70	95.65
C-I	0.14	28.00
O	0.82	17.99

## Conclusions

In this paper we have presented a two-phase hybrid approach to the segmentation of phenotype descriptions for the human skeletal phenome. The first phase performs a coarse-grained segmentation by splitting the description into its main anatomical and qualities entities, while the second phase focuses on a fine-grained segmentation within each category of entities. Experimental results have showed that, for phase I, without using domain dictionaries the best hybrid method can achieve the best F-Score of 96.90%, score that can be improved to 97.05% by adding the support of such dictionaries. Similar results have also been achieved in the second phase, i.e., 97.16% F-Score for the Anatomy category and 94.50% for the Quality category. Overall, our experiments lead to the conclusion that using an ensemble of classifiers for segmentation tasks may not necessarily improve the overall accuracy because of its dependency on the goal and underlying data characteristics.

While the research presented in this paper has been motivated by the SKELETOME project, the resulting classifiers can be used in any application scenario that requires as input decomposed skeletal phenotypes. Two main areas that can take immediate advantage of our results are ontology alignment and population – focused on aligning phenotype ontologies and populating phenotype ontologies with instances mined from the literature –, and building exploratory and educational tools in the context of skeletal diseases, using gamuts as input data.

## Endnote

^a^Bone dysplasias are a group of heterogeneous genetic disorders that affect predominantly the skeletal development. Patients diagnosed with such disorders suffer from complex medical issues that can be described via clinical findings, e.g., pains in limbs, radiographic findings, e.g., bilateral arachnodactyly and genetic findings, e.g., deletion mutation in FGFR3.

## Competing interests

The authors declare that they have no competing interests.

## Authors’ contributions

TG, AZ annotated the corpora. AZ provided the domain expertise required for the manual segmentation. TG developed the methods, performed the experiments. TG and JH interpreted the results. TG, JH and AZ wrote the paper. All authors read and approved the final manuscript.

## Supplementary Material

Additional file 1 Appendix. Click here for file
