# Supplementary material for: Supervised segmentation of phenotype descriptions for the human skeletal phenome using hybrid methods
Source: BMC Bioinformatics. 2012 Oct 15;13:265. doi: 10.1186/1471-2105-13-265 (PMC3495645; doi:10.1186/1471-2105-13-265)
Supplement: Additional file 1 — Appendix. [file 1471-2105-13-265-S1.pdf]

Appendix

Table 1 - Segmentation results for the individual classifiers with the use of domain dictionaries.

Model	P (%)	R (%)	F-1 (%)
CRF++	96.56	96.56	96.56
MALLET	96.24	96.24	96.24
YamCha1vs1	96.89	96.89	96.89
YamCha1vsAll	96.98	96.98	**96.98**

Table 2 - Segmentation results for the individual classifiers without the use of domain dictionaries.

Model	P (%)	R (%)	F-1 (%)
CRF++	96.26	96.26	96.26
MALLET	95.97	95.97	95.97
YamCha1vs1	96.76	96.76	96.76
YamCha1vsAll	96.80	96.80	**96.80**

Table 3 - Top segmentation results for paired and combined set operations, with domain dictionaries.

Method	P (%)	R (%)	F-1 (%)
YamCha1vs1 ∪ YamCha1vsAll	96.52	97.18	**96.85**
CRF++ ∪ YamCha1vsAll	95.96	97.44	96.70
YamCha1vs1 ∩ YamCha1vsAll	96.69	96.69	96.69
MALLET ∪ YamCha1vsAll	95.18	97.87	96.51
(MALLET ∪ YamCha1vs1) ∩ (CRF++ ∪ YamCha1vsAll)	96.62	97.16	96.89
(MALLET ∪ YamCha1vsAll) ∩ (YamCha1vs1 ∪ CRF++)	96.64	97.17	**96.91**
(MALLET ∩ CRF++) ∪ (YamCha1vs1 ∩ YamCha1vsAll)	96.64	97.08	96.86

Table 4 - Top segmentation results for paired and combined set operations, without domain dictionaries.

Method	P (%)	R (%)	F-1 (%)
YamCha1vs1 ∪ YamCha1vsAll	96.31	97.11	**96.70**
CRF++ ∪ YamCha1vsAll	95.21	97.70	96.44
YamCha1vs1 ∩ YamCha1vsAll	96.45	96.45	96.45
MALLET ∪ YamCha1vsAll	94.93	97.62	96.25
(MALLET ∪ YamCha1vs1) ∩ (CRF++ ∪ YamCha1vsAll)	96.22	97.11	96.66
(MALLET ∪ YamCha1vsAll) ∩ (YamCha1vs1 ∪ CRF++)	96.23	97.12	**96.67**
(MALLET ∩ CRF++) ∪ (YamCha1vs1 ∩ YamCha1vsAll)	96.22	96.99	96.60

Table 5 - Segmentation results for the voting mechanism with domain dictionaries.

Veto	P (%)	R (%)	F-1 (%)
CRF++	96.96	96.96	96.96
MALLET	96.94	96.94	96.94
YamCha1vs1	96.94	96.94	96.94
YamCha1vsAll	97.05	97.05	**97.05**

Table 6 - Segmentation results for the voting mechanism without domain dictionaries.

Veto	P (%)	R (%)	F-1 (%)
CRF++	96.60	96.60	96.60
MALLET	96.68	96.68	96.68
YamCha1vs1	96.90	96.90	**96.90**
YamCha1vsAll	96.90	96.90	**96.90**

Table 7 - Segmentation results for the individual classifiers for the Anatomy category.

Model	P (%)	R (%)	F-1 (%)
CRF++	97.11	97.11	**97.11**
MALLET	96.30	96.30	96.30
YamCha1vs1	96.90	96.90	96.90
YamCha1vsAll	96.94	96.94	96.94

Table 8 - Segmentation results for the individual classifiers for the Quality category.

Model	P (%)	R (%)	F-1 (%)
CRF++	92.34	92.34	92.34
MALLET	91.47	91.47	91.47
YamCha1vs1	94.07	94.07	94.07
YamCha1vsAll	94.50	94.50	**94.50**

Table 9 - Top segmentation results for paired and combined set operations the Anatomy category.

Method	P (%)	R (%)	F-1 (%)
MALLET ∪ CRF++	95.61	97.64	96.61
CRF++ ∪ YamCha1vs1	96.22	97.73	**96.97**
YamCha1vs1 ∪ YamCha1vsAll	96.78	97.03	96.90
YamCha1vs1 ∩ YamCha1vsAll	96.81	96.81	96.81
(MALLET ∪ YamCha1vs1) ∩ (CRF++ ∪ YamCha1vsAll)	96.66	97.29	96.98
(MALLET ∪ YamCha1vsAll) ∩ (YamCha1vs1 ∪ CRF++)	96.71	97.38	**97.04**
(MALLET ∩ CRF++) ∪ (YamCha1vs1 ∩ YamCha1vsAll)	96.70	97.29	97.00

Table 10 - Top segmentation results for paired and combined set operations the Quality category.

Method	P (%)	R (%)	F-1 (%)
MALLET ∪ CRF++	88.48	96.02	92.09
CRF++ ∪ YamCha1vs1	91.07	95.58	93.27
YamCha1vs1 ∪ YamCha1vsAll	93.84	94.64	**94.24**
YamCha1vs1 ∩ YamCha1vsAll	93.93	93.93	93.93
(MALLET ∪ YamCha1vs1) ∩ (CRF++ ∪ YamCha1vsAll)	93.11	94.72	93.91
(MALLET ∪ YamCha1vsAll) ∩ (YamCha1vs1 ∪ CRF++)	93.18	94.93	**94.05**
(MALLET ∩ CRF++) ∪ (YamCha1vs1 ∩ YamCha1vsAll)	93.10	94.57	93.83

Table 11 - Segmentation results for the voting mechanism the Anatomy category.

Veto	P (%)	R (%)	F-1 (%)
CRF++	97.16	97.16	**97.16**
MALLET	97.16	97.16	**97.16**
YamCha1vs1	96.94	96.94	96.94
YamCha1vsAll	96.94	96.94	96.94

Table 12 - Segmentation results for the voting mechanism the Quality category.

Veto	P (%)	R (%)	F-1 (%)
CRF++	94.28	94.28	94.28
MALLET	93.92	93.92	93.92
YamCha1vs1	94.22	94.22	94.22
YamCha1vsAll	94.50	94.50	**94.50**
